# The “DOLPHINS” Project: A Low-Cost Real-Time Multivariate Process Control From Large Sensor Arrays Providing Sparse Binary Data

**DOI:** 10.3389/fchem.2021.734132

**Published:** 2021-09-03

**Authors:** Eugenio Alladio, Marcello Baricco, Vincenzo Leogrande, Renato Pagliari, Fabio Pozzi, Paolo Foglio, Marco Vincenti

**Affiliations:** ^1^ Dipartimento di Chimica, Università Degli Studi di Torino, Torino, Italy; ^2^ RADA Snc–Soluzioni Informatiche, Rivoli, Italy; ^3^ CNH Industrial–Lungo Stura Lazio, Torino, Italy

**Keywords:** predictive maintenance, machine learning, sparse binary data, multivariate data analysis, principal component analysis, soft independent modeling by class analogy

## Abstract

The “DOLPHINS” project started in 2018 under a collaboration between three partners: CNH Industrial Iveco (CHNi), RADA (an informatics company), and the Chemistry Department of the University of Turin. The project’s main aim was to establish a predictive maintenance method in real-time at a pilot plant (CNHi Iveco, Brescia, Italy). This project currently allows maintenance technicians to intervene on machinery preventively, avoiding breakdowns or stops in the production process. For this purpose, several predictive maintenance models were tested starting from databases on programmable logic controllers (PLCs) already available, thus taking advantage of Machine Learning techniques without investing additional resources in purchasing or installing new sensors. The instrumentation and PLCs related to the truck sides’ paneling phase were considered at the beginning of the project. The instrumentation under evaluation was equipped with sensors already connected to PLCs (only on/off switches, i.e., neither analog sensors nor continuous measurements are available, and the data are in sparse binary format) so that the data provided by PLCs were acquired in a binary way before being processed by multivariate data analysis (MDA) models. Several MDA approaches were tested (e.g., PCA, PLS-DA, SVM, XGBoost, and SIMCA) and validated in the plant (in terms of repeated double cross-validation strategies). The optimal approach currently used involves combining PCA and SIMCA models, whose performances are continuously monitored, and the various models are updated and tested weekly. Tuning the time range predictions enabled the shop floor and the maintenance operators to achieve sensitivity and specificity values higher than 90%, but the performance results are constantly improved since new data are collected daily. Furthermore, the information on where to carry out intervention is provided to the maintenance technicians between 30 min and 3 h before the breakdown.

## Introduction

The current and future sustainable economic growth of companies worldwide are today, more than ever, increasingly based on the value and the information created by data. In the field of industry, the features of Industry 4.0 are showing a growing impact on the productive processes, since the companies are financially encouraged to move towards industrial automation that integrates some new production technologies aimed at improving working conditions, creating new business models, and increasing the productivity and product quality of their plants. Furthermore, the governments of several countries are promoting business plans and strategies focused on Industry 4.0 to offer the companies the tools aimed at seizing the opportunities of innovation and digital instruments related to the current fourth industrial revolution (Gentner, 2016; Enyoghasi and Badurdeen, 2021; Gallo et al., 2021; Ghobakhloo et al., 2021). In this context, Big Data and Data Analytics themes play a strategic role since data are indeed considered the lifeblood of economic development of the industrial (but not only) companies nowadays. Data are the basis for evaluating the quality of the products and generating gains in productivity and resource efficiency, making it possible to optimize the production process and enhance the whole plant’s efficiency. Consequently, many companies face the necessity of implementing strategies capable of collecting and interpreting the data robustly and systematically alongside their productive process (Cugno et al., 2021; Goldman et al., 2021; Jeske et al., 2021; Lee and Lim, 2021). Various Multivariate Data Analysis (MDA) models and Machine Learning (ML) approaches have been gradually introduced within the production plants to develop competitive strategies, such as process control, quality control, and predictive maintenance (Elsisi et al., 2021; Jamwal et al., 2021; Lee and Lim, 2021; Wankhede and Vinodh, 2021). The last topic is fascinating for the companies since, if historical data have been already stored in databases, predictive maintenance substantially requires the computation of ML algorithms to predict the necessity of a repair or, eventually, a replacement, which can be therefore programmed and performed the way it turns to be most effective. Predictive maintenance was originally performed using user-defined alerts or expert-defined thresholds involving Supervisory Control And Data Acquisition (SCADA) systems. However, this approach does not consider the presence of correlations, patterns, and similarities among the collected features and the available signals detected from the sensors on the machinery. On the other hand, MDA and ML tools perform a multivariate interpretation of the stored data, which can belong to even different kinds of databases (e.g., sensors, SCADA, and history data) and origins (e.g., IT data, shop floor information, and manufacturing processes) (Ghobakhloo et al., 2021; Lee and Lim, 2021). The current work focuses on developing and testing several Machine Learning approaches at a pilot automotive plant (CNHi Iveco, Brescia, Italy) for predictive maintenance purposes. In particular, the goal of the “DOLPHINS” project was to build a low-cost edge digital twin capable of performing real-time predictive maintenance starting from data already collected and available at the plant level. This project was settled in 2018 under a collaboration among CNH Industrial Iveco (CHNi), RADA (an informatics company), and the Department of Chemistry of the University of Turin. In more detail, the goal of the DOLPHINS project was to develop a software application—in the tangible form of a dashboard working in real-time as a statistical digital twin of a shopfloor asset—by implementing a twin statistical model of the equipment under examination to deliver behavioral predictive warnings to the maintenance technicians in order to intervene on the investigated machinery preventively. Fundamental targets of the DOLPHINS project were as follows: 1) to provide the technicians an approach showing robust predictive capabilities of performing real-time maintenance; 2) to diminish as much as possible the occurrences of breakdowns, stops, and micro-stops, aspiring to a near-zero downtime goal; 3) to develop a low-cost implementation of this approach since training data for ML and MDA approaches were already collected and stored in programmable logic controller (PLC) devices. By referring to the last DOLPHINS target, a relevant advantage of this project is that ML models were built on data already available by the PLC equipment itself from large sensor arrays. Hence, no additional sensors were needed since the multivariate models were trained on the historical data and then tested on those acquired recently, reducing the impact on the company in terms of implementation costs and time. Since data are stored by the PLCs in the form of sparse binary matrices, several ML algorithms were tested during the development stage of DOLPHINS. Therefore, various MDA classification models were evaluated to predict the occurrence of failures within a given time window and their performance was monitored to choose the optimal model to perform constant and real-time processing of the data. Finally, once new data and signals are collected, they are interpreted by the developed ML model to monitor the performance of the machinery under examination and predict its evolution by detecting any significant drift and variation over time. The real-time results are expressed in terms of the probability of malfunctions and severity of the signals recorded by the PLCs to allow the maintenance technicians to work promptly on specific machinery sections. This approach diminishes the occurrences of stops and breakdowns sensitively and provides further knowledge on the behavior of the machinery itself. The final goal of the DOLPHINS project is to extend this approach to other shopfloor systems by raising the amount of cost savings, diminishing the periods of downtime, and improving the efficiency and the predictability of the productive process.

## Materials and Methods

### Framework and Project Development

**FIGURE 1:**
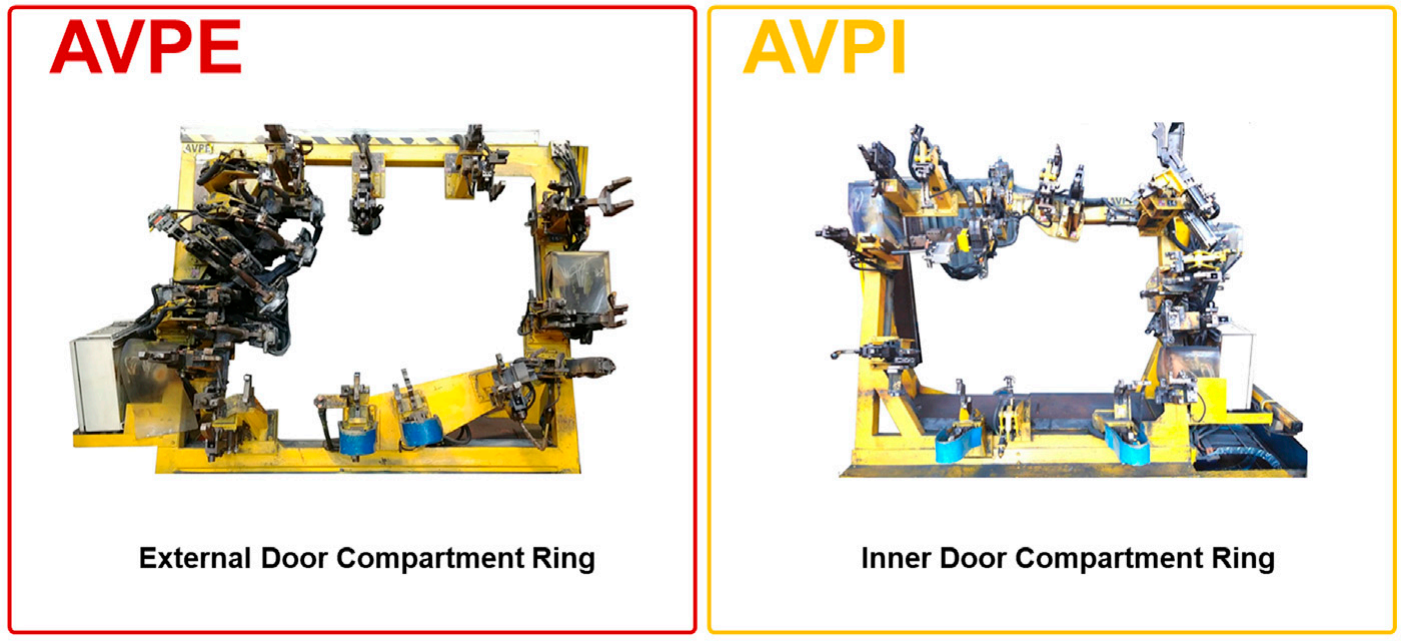
Graphical representation of the External Door Compartment Ring (AVPE) and Inner Door Compartment Ring (AVPI) machinery under examination of the CNH Industrial Iveco (CHNi) Plant of Brescia (Italy).

The development of the DOLPHINS project started as a proof-of-concept study on evaluating the data acquired at the CNH Industrial Iveco (CHNi) Plant of Brescia (Italy). The working area selected for the project consisted of the Welding Operative Unit and two types of machinery were monitored [namely, 0P10–External Door Compartment Ring (AVPE) and 0P10–Internal Door Compartment Ring (AVPI)]. Signals registered from AVPE and AVPI machinery (Figure 1) were historically stored into PLCs but not interpreted in ML modeling for predictive maintenance. In detail, the data of AVPE and AVPI consisted of 176 and 153 sensors connected to the PLCs, respectively. The signals were registered into the database in the form of sparse binary output (i.e., ON/OFF, with a prevalence of OFF results), indicating if the specific threshold values for each sensor are exceeding (i.e., ON) or not (i.e., OFF). Then, a pre-treatment step involving the binarization of the collected data (i.e., 0 means that the signal of a specific collected variable is OFF, whereas 1 means that the signal of the variable is ON) was performed. Furthermore, a categorical binary output indicating the status of the machinery (i.e., “Working” or “Stop”) for each collection record was available, too.

**FIGURE 2:**
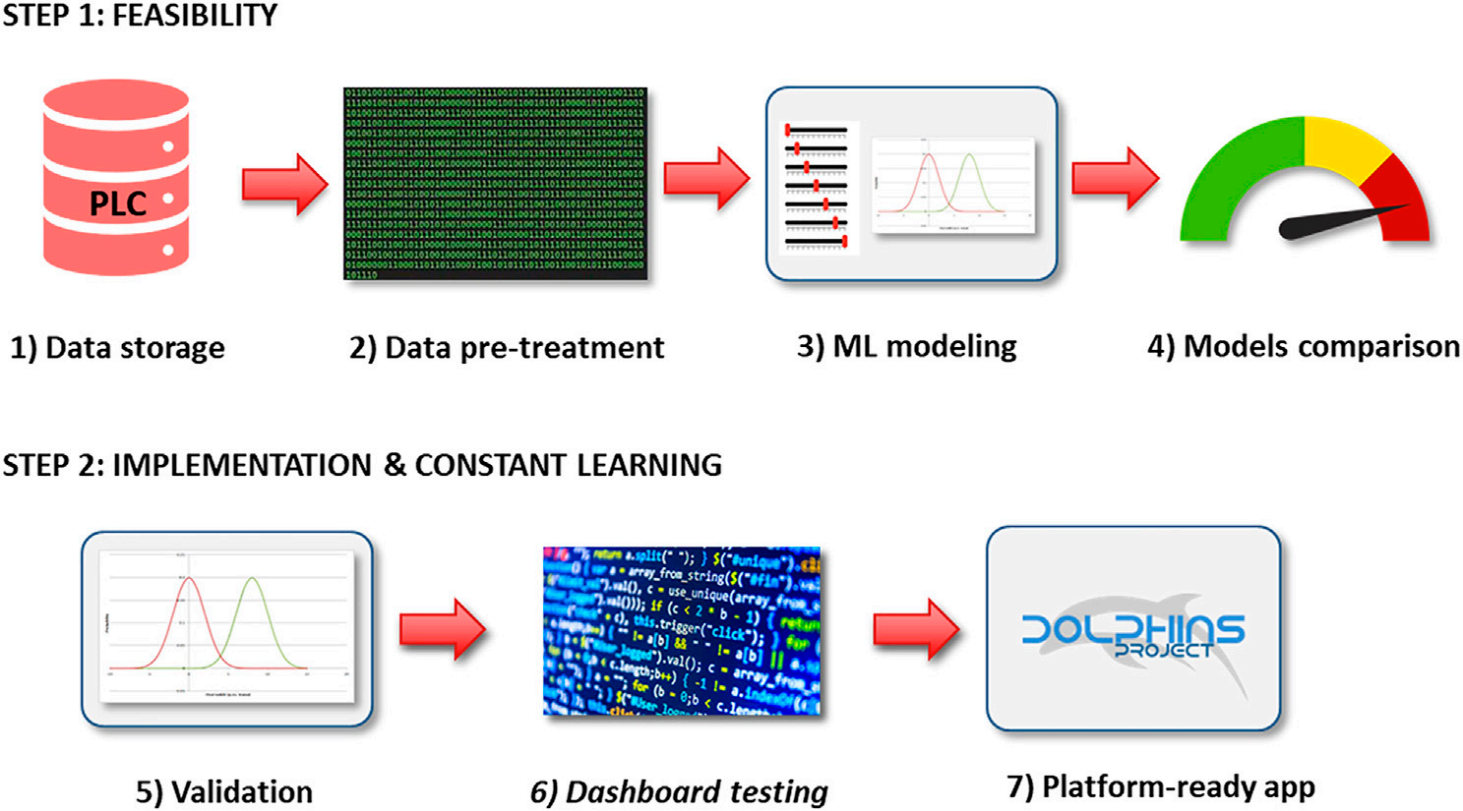
Working steps of the DOLPHINS project.

In the present proof-of-concept study, the records collected from September 1, 2020, up to November 15, 2020, are shown as an example of two matrices of dimensions 210,307 × 177 and 199,077 × 154 for AVPE and AVPI machinery, respectively. Records are collected on the PLCs with a frequency of one second per record during the different work shifts. The whole study was composed of two developmental steps: the first step assessed the feasibility of the study, involving the acquisition of the data, their pre-pretreatment, the evaluation of several ML models, and the comparison of their performances, while the second step focused on the real-time implementation of the developed model within the plant, by testing the elected ML model on newly acquired data, updating the model with a scheduled frequency (approx. one month), and programming dashboards and platform-ready applications to be employed by the maintenance technicians during their everyday work. A graphical representation of the developmental steps of the DOLPHINS project is reported in Figure 2.

### Machine Learning Strategies

Several classification ML models were tested on the collected data to decide which algorithm best discriminates the records labeled as “Working” conditions from those labeled as “Stop” conditions of both the AVPE and the AVPI machinery. For this purpose, a benchmark analysis was performed by involving the following classification algorithms: k-Nearest Neighbors (kNN) (Massart et al., 1997), Logistic Regression (LogReg) (Cruyff et al., 2016), Linear Discriminant Analysis (LDA) (Massart et al., 1997; Martinez and Kak, 2001), Quadratic Discriminant Analysis (QDA) (Srivastava et al., 2007), Partial Least Squares–Discriminant Analysis (PLS-DA) (Ballabio and Consonni, 2013), Soft Independent Modelling of Class Analogies (SIMCA) (Wold and Sjostrom, 1977; Vanden Branden and Hubert, 2005), Naive Bayes (NB) (Cassidy, 2020), Support Vector Machine (SVM) (Hearst et al., 1998; Vapnik, 2000), Decision Trees (DT), Random Forest (RF) (Fratello and Tagliaferri, 2019), and Extreme Gradient Boosting (XGBoost) (Chen and Guestrin, 2016; Guang et al., 2020). Since the acquired data are in the form of sparse binary matrices, the Sparse Logistic Principal Components Analysis (SL-PCA) approach was performed on the datasets before computing different ML approaches such as kNN, LogReg, LDA, QDA, and SIMCA. Since these algorithms can not evaluate sparse binary data properly, they were calculated on the Principal Components (PCs) provided by SL-PCA modeling. The SL-PCA algorithm introduced by Lee et al. (2010) involves an iterative weighted least squares algorithm and the calculated PCs were then used as new variables for the cited ML algorithms.

Both external validation and cross-validation were performed in the study. All the MDA models were tuned and trained on the data from September 1, 2020, up to October 31, 2020, using a repeated k-fold cross-validation strategy. For benchmark and tuning purposes, each tested algorithm got the same training set since the data had the same partitioning for every model and every cross-validation step. This data partitioning strategy was employed to compare the performance of the various models properly.

Grid search analysis was made to tune the number of components and the values of the hyperparameters of all the algorithms effectively. The use of an exhaustive grid search analysis (involving cross-validation, too) was performed to find the combination of hyperparameters that performed best for each ML model. Grid search analysis (rather than random search or sequential search) allowed us to monitor many values within the hyperparameters’ space when looking for the best-performing values. Despite grid search being time-consuming and expensive, we decided to exploit it to achieve the best tuned and cross-validated ML models. SL-PCA tuning grid search involved evaluating the optimal number of *k* components (from 1 up to 30) and λ penalty parameter (from 0 up to 0.01). The best compromise for the goodness-of-fit and the model complexity was achieved by minimizing the Bayesian Information Criterion (BIC) (Lan et al., 2012). Grid search was performed for PLS-DA and SIMCA to find the optimal number of *k* components and latent variables in terms of Root Mean Square Error in Cross-Validation (RMSECV) (Massart et al., 1997). The optimal value of *k-*nearest neighbors for the kNN algorithm was varied from 1 up to 10. No tuning grid search was required for LDA, QDA, LogReg, and NB algorithms. In contrast, SVM tuning involved the grid search evaluation of four hyperparameters: *kernel* (involving the use of polynomial, radial, or sigmoid kernels), *degree* (related to the shape of the SVM decision boundaries for polynomial kernels, from 1 up to 3), *gamma* (describing the influence of the records on the location of the SVM decision boundaries, from 0.1 up to 10), and *C* (influencing the penalization of the records arranged within the margin of SVM boundary, from 0.1 up to 10) (Vapnik, 1995). DT tuning involved the grid search approach on four hyperparameters: *minsplit* (describing the minimum amount of records to be included into a node before splitting, from 1 up to 20), *minbucket* (defining the maximum depth of the calculated decision tree, from 1 up to 10), *cp* (indicating the minimum improvement in the performance of a node to allow a further split, from 0.01 up to 0.1), and *maxdepth* (describing the minimum amount of records that can be included into a leaf, from 1 up to 10). RF algorithm also involved the grid search tuning evaluation of four hyperparameters: *ntree* (expressing the number of trees in the forest model, from 10 up to 300), *mtry* (representing the number of variables to be randomly sampled at each node, from 5 up to 40), *nodesize* (defining the minimum number of records to be included into a node, 1 up to 10), and *maxnodes* (establishing the maximum number of leaves allowed in the model, from 2 up to 30) (Bischl et al., 2016; Fratello and Tagliaferri, 2019). XGBoost tuning grid search evaluated seven hyperparameters: *eta* (indicating the learning rate to avoid overfitting, from 0 up to 1), *gamma* (describing the minimum amount of splitting for a node, from 0 up to 20), *max_depth* (indicating how deeply each evaluated tree can grow, from 1 up to 5), *min_child_weight* (defining the level of impurity that is maintainable for a node, from 1 up to 10), *subsample* (describing the proportion of samples to be randomly selected when evaluating each tree, from 0 up to 1), *colsample_bytree* (evaluating the proportion of variables selected by each tree, from 0.1 up to 1), and *nrounds* (defining the number of trees that can be sequentially calculated within the model, from 10 up to 100) (Bischl et al., 2016; Guang et al., 2020). The tuning of the kNN, SVM, DT, RF, and XGBoost methods was evaluated in terms of mean misclassification error (MMCE), which represents the ratio between the number of records classified as belonging to a specific class different from their actual class (i.e., “Stop” or “Working”) (Bischl et al., 2016; Probst et al., 2017). This parameter was calculated for all the ML algorithms and, therefore, the best tuning scenarios selected turned to be those providing the lowest MMCE value. A repeated k-fold cross-validation strategy involving a 10-fold CV approach repeated five times was performed when performing the grid search analysis. As a result, in summary, the best models were selected in average terms of Bayesian Information Criterion (BIC) for SL-PCA, Root Mean Square Error in Cross-Validation (RMSECV) for SIMCA and PLS-DA, and mean misclassification error (MMCE) for kNN, SVM, DT, RF, and XGBoost. This approach, in our opinion, validates our entire model-building procedure, including the hyperparameter-tuning step.

The external validation was made by removing the records from November 1, 2020, up to November 15, 2020 from AVPE and AVPI original datasets. These data, consisting of matrices of dimensions 42,062 × 177 for AVPE and 33,180 × 154 for AVPI, were employed as a test set. Therefore, the results and the performance of the ML algorithms on the external validation test set were expressed using several metrics such as precision, recall, specificity, and F_score_. In the present studio, the records classified as “Working” were considered positive samples (i.e., indicating proper functioning of the tested machinery). In contrast, the records classified as “Stop” were considered negative samples (i.e., indicating a malfunction or a breakdown of the machinery under examination). The “models” performance metrics were calculated as follows:

$$Precision=\frac{TP}{TP+FP},$$

$$Recall=\frac{TP}{TP+FN},$$

$$Specificity=\frac{TN}{TN+FP},$$

$$F_{score}=\frac{2\times Recall\times Precision}{Recall+Precision},$$

where TP and FP represent the number of true positive and false positive records, whereas TN and FN indicate the number of true negative and false-negative records (Bischl et al., 2016). Finally, the model showing the best compromise among the different performance metrics was employed to develop the dashboards for predictive maintenance and the real-time evaluation of the new data collected in the plant. Nevertheless, an update of the ML models was scheduled with a frequency of 1 month (in parallel with the real-time analysis of the new data) to monitor the performance of the ML models on a larger amount of data.

### Software

R statistical environment (version 4.0.2) (R Core Team, 2020) and R Studio Desktop IDE (version 1.4.1717) (RStudio Team, 2020) were used in this study. In addition, the following R packages were employed: *caret* (Kuhn, 2020), *dplyr* (Wickham et al., 2020), *ggplot2* (Wickham, 2016), *mdatools* (Kucheryavskiy, 2020), *mixOmics* (Rohart et al., 2017), *mlr* (Bischl et al., 2016), *parallel* (R Core Team, 2020), *parallelMap* (Bischl et al., 2020), *plotly* (Sievert, 2018), and *tidyverse* (Wickham et al., 2019). PLS-DA modeling was performed using the R codes available at (Github, 2013).

## Results and Discussion

### Tuning and Benchmark Analysis

**FIGURE 3:**
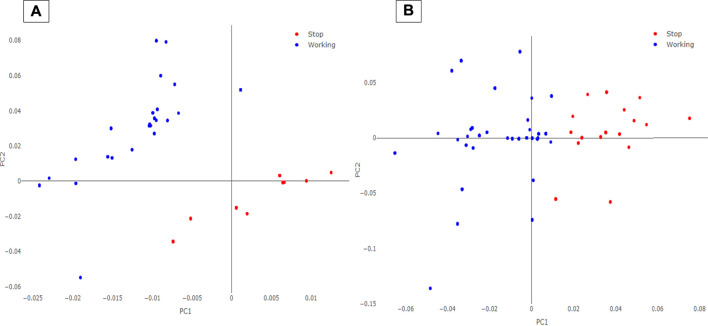
SL-PCA scores plot for AVPE **(A)** and AVPI **(B)** machinery. The blue circles represent the records labeled as “Working” on the PLCs, while the red circles are the records acquired as “Stop.”

SL-PCA modeling indicated, as optimum, a tuning of 6 PCs with a λ value of the penalty parameter equal to 0.0025 for the AVPE data 5 PCs and λ equal to 0.0020 for the AVPI data. Examples of the scores plots of the SL-PCA models on the AVPE and AVPI machinery training datasets are reported in Figure 3. As it can be seen, a distinct separation is observed between the “Working” and the “Stop” samples in the space modeled by the new PCs. Since PCA is an exploratory data analysis algorithm, these results suggest that the classification task focused on predicting the operative conditions and the behaviors of the machinery under examination might be feasible. Therefore, the calculated PCs were used as new features for the following ML classification algorithms: kNN, LogReg, LDA, QDA, and SIMCA.

The results for all the evaluated ML algorithms are expressed in MMCE for AVPE and AVPI machinery in Table 1. Further details about the tuning results for all the models are reported in the Supplementary Material (Supplementary Table S1). As shown in Table 1, SIMCA modeling (preceded by SL-PCA processing) provided the lowest results in MMCE. Therefore, this approach was selected for further testing with the external validation data and the implementation within an on-purpose developed dashboard to be used at the shopfloor level by the maintenance technicians of the plant.

**TABLE 1:** MMCE values of all the tested ML algorithms for AVPE and AVPI machinery training datasets.

ML models	AVPE (MMCE)	AVPI (MMCE)
KNN	0.073	0.075
LogReg	0.114	0.093
LDA	0.099	0.127
QDA	0.085	0.106
PLS-DA	0.171	0.216
SIMCA	0.034	0.052
NB	0.210	0.194
SVM	0.052	0.081
DT	0.226	0.102
RF	0.083	0.135
XGBoost	0.097	0.143

### SIMCA Model

**FIGURE 4:**
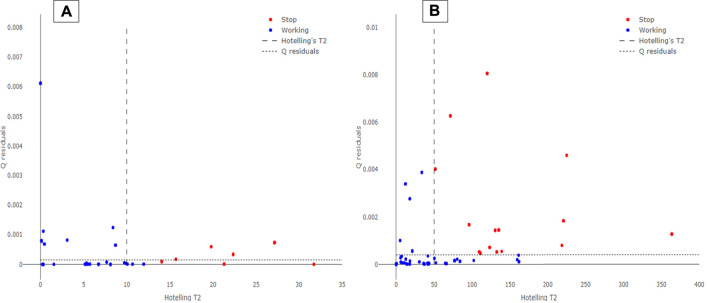
Hotelling’s T^2^ vs. Q residuals plot for AVPE **(A)** and AVPI **(B)** machinery. The blue circles represent the records labeled as “Working” on the PLCs, while the red circles are the records acquired as “Stop.” The dotted line indicates the 95% Hotelling’s T^2^ limit, while the dashed line represents the 95% Q residuals limit.

The external validation dataset involving the AVPE and the AVPI data from November 1, 2020, up to November 15, 2020, were predicted by the developed SIMCA model. Hotellings T^2^ vs. Q residuals plots for AVPE and AVPI test sets can be observed in Figure 4. Again, a satisfactory separation is observed between the “Working” and the “Stop” records collected by the PLCs during the period under examination. Although some records are still misclassified (mainly false negatives, i.e., false “Stop” predictions), the performance of the SIMCA model appears robust for both AVPE and AVPI machinery, thus suggesting the use of this approach for predictive maintenance purposes.

SIMCA prediction results are expressed in precision, recall, specificity, and F_score_ for both the types of machinery under examination, as reported in Table 2. These evaluations were made for all the ML algorithms, but the results turned to be lower than those obtained by the SIMCA model (results not reported here). SIMCA model provided optimal results for all the metrics under examination. However, specificity turned to be the metric with the lowest value; this result may be due to the lower number of “Stop” occurrences collected by the PLCs. The machinery under examination does not stop frequently, and several recorded “Stop” instances can be defined as micro-stops since they show a downtime lower than 1 minute. Moreover, the number of “Stop” records collected by the PLCs is only around 5% of the data. Our opinion is that the model’s performance might be improved further by updating the training sets in a scheduled way (approx. one month) and collecting new data, especially those related to “Stop” records. Since the approach involving SL-PCA and SIMCA algorithms provided optimal and robust classification performance, this method was implemented into a dashboard to perform real-time predictive maintenance in the plant.

**TABLE 2:** SIMCA performance metrics for AVPE and AVPI machinery test datasets.

Machinery	Precision	Recall	Specificity	F_SCORE_
AVPE	0.977	0.944	0.844	0.960
AVPI	0.985	0.962	0.899	0.973

### Dashboard Implementation

SIMCA algorithm provides several advantages for the development of a real-time predictive maintenance approach. Firstly, no assumptions are made on the probability distributions of the features under examination, which allows analyzing the new PCs from the SL-PCA model reliably. Secondly, since each class (i.e., “Working” and “Stop” records) is modeled independently, it is possible to obtain and predict the information about the classification probability of a certain record when introduced into the trained model. Thirdly, the SIMCA approach allows the maintenance technicians to identify the sensor or the single component of the machinery to consider for intervention before the occurrence of an incoming fault. Thanks to the evaluation of Hotelling’s T^2^ and Q residuals contribution plots provided by the combined SL-PCA and SIMCA algorithms, it is possible to recognize the critical signals recorded on the PLCs. An indication of the severity of the recorded signals was also implemented by calculating the logarithm (base 10) of the maximum Hotelling’s T^2^ and Q residuals contribution (in absolute value) and normalizing it on a scale from 0 up to 100%. However, this approach is still under evaluation since the amount of recorded breakdown events is relatively low.

**FIGURE 5:**
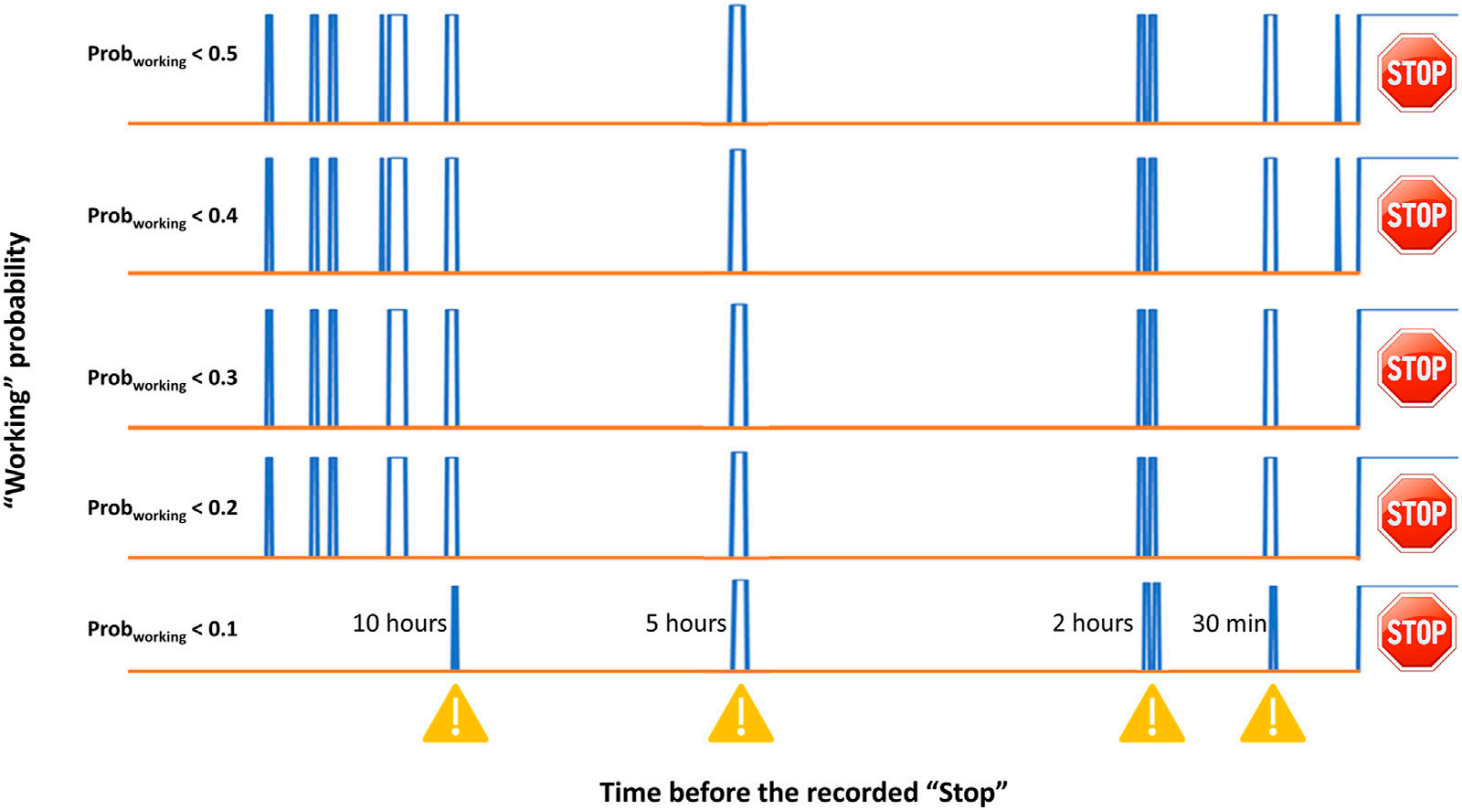
Transient of SIMCA Prob_working_ monitored over the time for the AVPE machinery before a breakdown. The x-axis represents the time before a breakdown event (occurring on the right side of the plot), while the blue lines represent the coded transient at different Prob_working_ thresholds (i.e., 0.5, 0.4, 0.3, 0.2 and 0.1).

Finally, the probability of classifying a new record as “Working” (Prob_working_) is inferred for all the new records collected on the PLCs. Figure 5 displays the transient of Prob_working_ over time. The example reported in Figure 5 shows the fluctuation of such probability before a specific breakdown occurred (on the right part of the plot). The x-axis represents the time before the occurrence of the stop of the machinery (in this case, AVPE), while the y-axis shows a binary output related to Prob_working_. As a rule of thumb, it was established that if Prob_working_ turns higher (or equal) than 0.5, the record is classified as “Working” and the transient is set to 0. On the other hand, if Prob_working_ turns to be lower than 0.5, the record is classified as “Stop” and the transient is set to 1. The indication of a probable malfunction of the AVPE machinery was observed, in this case, 10 h before the breakdown. Other alerts were predicted 5, 2 h, and 30 min before the adverse event. However, the number of false “Stop” occurrences (i.e., false-negative records) might be rather high, as also remarked by the specificity values reported in Table 2. Again, this might be ascribed to the necessity of collecting new data and updating the SIMCA models (or the other tested ML algorithms). Nevertheless, further tuning of the employed decoding was tested. As it can be seen in Figure 5, different thresholds of Prob_working_ were evaluated (e.g., 0.4, 0.3, 0.2, and 0.1 thresholds) to diminish the number of false “Stop” occurrences and improved sensitivity values (approx. 0.89 and 0.94 for AVPE and AVPI, respectively) were found using a Prob_working_ threshold of 0.1. This further refining of the algorithms is still under examination and will be monitored over time. Furthermore, this approach allows providing the maintenance technician a tool capable of predicting a breakdown event before its occurrence. In fact, by analyzing the transients of Prob_working_ monitored over time, it was observed that reliable alerts occurred in the range between 30 min and 3 h before the breakdown. At the current stage, it is still not trustworthy to provide Prob_working_ with a confidence interval in terms of time before the occurrence of the stop event since the number of “Stop” records is still limited. However, further analyses will be made on Prob_working_ over time to estimate such a parameter reliably. An example of the developed dashboard is shown in Supplementary Figure S2.

## Conclusion

DOLPHINS project represents a proof-of-concept and low-cost tool to perform reliable real-time predictive maintenance. It combines ML technology-driven algorithms with the evaluation of historical datasets that have never been interpreted using a multivariate data analysis approach. The algorithm involving SL-PCA and SIMCA has now been implemented by the automotive plant of CNHi Iveco (Brescia, Italy) at the shop floor level efficiently, and the number of failures and breakdown events has significantly diminished since the commissioning of the project.

This project allowed the development of an automated dashboard that shows the operator, in real-time, and the current instrumentation’s operating conditions and, if signals arrive at the PLC, indicates the severity and probability that these lead to a stop. This predictive maintenance approach has numerous advantages, including 1) a meager impact in terms of costs (data already available are used); 2) the possibility of physically interpreting the information; 3) the possibility of not having to stop the production process; 4) the transversality of the application of Machine Learning also to other components and instrumentation within the plant.

At the current stage, the DOLPHINS algorithm can run on edge and cloud systems and conventional plant infrastructure. For this reason, the future perspectives of this project will focus on converting the DOLPHINS algorithm into a multiplatform application to raise its scalability on other types of machinery and plants. However, DOLPHINS is now equipment-oriented, and all the steps involving the tuning, training, and testing of the ML algorithms are required to develop a robust real-time predictive maintenance strategy. Therefore, a constant and frequent update of the databases and the ML models have to be scheduled to obtain reliable results and reach the goal of near-zero downtime.

.

## Data Availability

The raw data supporting the conclusion of this article will be made available by the authors without undue reservation.
