# Supplementary material for: The “DOLPHINS” Project: A Low-Cost Real-Time Multivariate Process Control From Large Sensor Arrays Providing Sparse Binary Data
Source: Front Chem. 2021 Sep 3;9:734132. doi: 10.3389/fchem.2021.734132 (PMC8446282; doi:10.3389/fchem.2021.734132)
Supplement: Supplementary file 1 [file DataSheet1.PDF]

Table S1. Optimal values of the hyperparameters of the tested ML algorithms after the grid search tuning.

ML Models	Tuning Results
kNN	*k* = 7 (MMCE = 0.147)
PLS-DA	*k* = 6 (RMSECV = 12.384)
SVM	*kernel* = polynomial, *degree* = 2, *gamma* = 3, *C* = 2 (MMCE = 0.0935)
DT	*minsplit* = 4, *minbucket* = 3, *cp* = 0.25, *maxdepth* = 5 (MMCE = 0.119)
RF	ntree = 30, mtry = 10, nodesize = 7, maxnodes = 12 (MMCE = 0.0895)
XGBoost	*eta* = 0.2, *gamma* = 10, *max_depth* = 3, *min_child_weight* = 6, *subsample* = 0.5, *colsample_bytree* = 0.5, *nrounds* = 20 (MMCE = 0.0884)

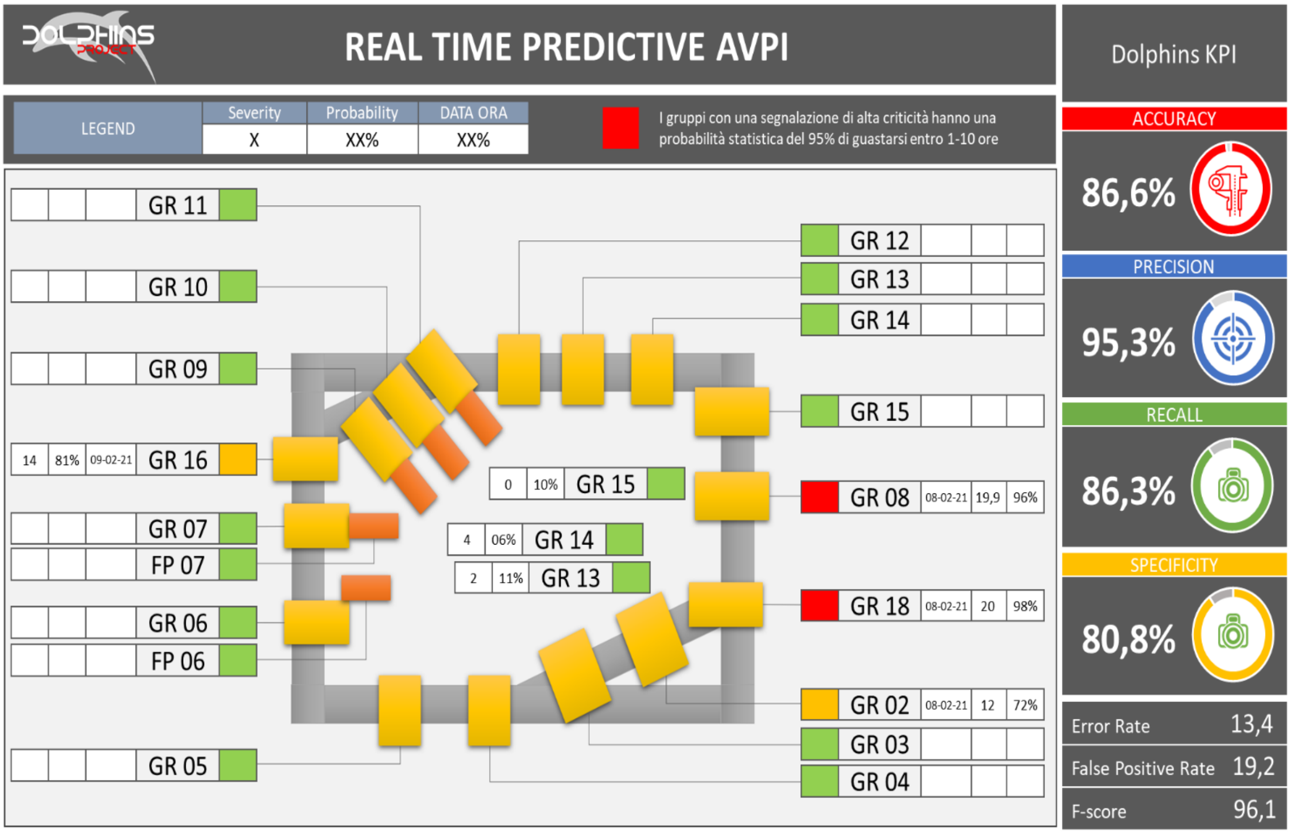

Figure S2. Example of the dashboard implemented at the automotive plant of CNHi Iveco (Brescia, Italy)
